# Altered surfactant homeostasis and recurrent respiratory failure secondary to TTF-1 nuclear targeting defect

**DOI:** 10.1186/1465-9921-12-115

**Published:** 2011-08-25

**Authors:** Donatella Peca, Stefania Petrini, Chryssoula Tzialla, Renata Boldrini, Francesco Morini, Mauro Stronati, Virgilio P Carnielli, Paola E Cogo, Olivier Danhaive

**Affiliations:** 1 Department of Medical and Surgical Neonatology, Bambino Gesù Children's Hospital IRCCS, Rome, Italy; 2 Research Center, Bambino Gesù Children's Hospital IRCCS, Rome, Italy; 3 Division of Neonatology, Fondazione-IRCCS Policlinico San Matteo, Pavia, Italy; 4 Division of Clinical Pathology, Bambino Gesù Children's Hospital IRCCS, Rome, Italy; 5 Neonatal Division, Institute of Maternal-Infantile Sciences, Polytechnic University of Marche, Azienda Ospedaliera Universitaria Ospedali Riuniti Ancona, Italy; 6 Pediatric Cardiosurgical Intensive Care Unit, Bambino Gesù Children's Hospital IRCCS, Rome, Italy

**Keywords:** thyroid transcription factor 1, ATP binding cassette transporters, lung diseases, interstitial, pulmonary surfactants, pituitary insufficiency, pulmonary surfactant-associated protein B, lung-brain-thyroid syndrome

## Abstract

**Background:**

Mutations of genes affecting surfactant homeostasis, such as *SFTPB*, *SFTPC *and *ABCA3*, lead to diffuse lung disease in neonates and children. Haploinsufficiency of *NKX2.1*, the gene encoding the thyroid transcription factor-1 (TTF-1) - critical for lung, thyroid and central nervous system morphogenesis and function - causes a rare form of progressive respiratory failure designated brain-lung-thyroid syndrome. Molecular mechanisms involved in this syndrome are heterogeneous and poorly explored. We report a novel TTF-1 molecular defect causing recurrent respiratory failure episodes in an infant.

**Methods:**

The subject was an infant with severe neonatal respiratory distress syndrome followed by recurrent respiratory failure episodes, hypopituitarism and neurological abnormalities. Lung histology and ultrastructure were assessed by surgical biopsy. Surfactant-related genes were studied by direct genomic DNA sequencing and array chromatine genomic hybridization (aCGH). Surfactant protein expression in lung tissue was analyzed by confocal immunofluorescence microscopy. For kinetics studies, surfactant protein B and disaturated phosphatidylcholine (DSPC) were isolated from serial tracheal aspirates after intravenous administration of stable isotope-labeled ^2^H_2_O and ^13^C-leucine; fractional synthetic rate was derived from gas chromatography/mass spectrometry ^2^H and ^13^C enrichment curves. Six intubated infants with no primary lung disease were used as controls.

**Results:**

Lung biopsy showed desquamative interstitial pneumonitis and lamellar body abnormalities suggestive of genetic surfactant deficiency. Genetic studies identified a heterozygous *ABCA3 *mutation, L941P, previously unreported. No *SFTPB*, *SFTPC *or *NKX2.1 *mutations or deletions were found. However, immunofluorescence studies showed TTF-1 prevalently expressed in type II cell cytoplasm instead of nucleus, indicating defective nuclear targeting. This pattern has not been reported in human and was not found in two healthy controls and in five *ABCA3 *mutation carriers. Kinetic studies demonstrated a marked reduction of SP-B synthesis (43.2 vs. 76.5 ± 24.8%/day); conversely, DSPC synthesis was higher (12.4 vs. 6.3 ± 0.5%/day) compared to controls, although there was a marked reduction of DSPC content in tracheal aspirates (29.8 vs. 56.1 ± 12.4% of total phospholipid content).

**Conclusion:**

Defective TTF-1 signaling may result in profound surfactant homeostasis disruption and neonatal/pediatric diffuse lung disease. Heterozygous ABCA3 missense mutations may act as disease modifiers in other genetic surfactant defects.

## Introduction

Genetic disorders of surfactant homeostasis are a rare cause of respiratory failure in newborns and infants [1]. Bi-allelic loss-of-function mutations of *SFTPB*, the gene encoding surfactant protein-B (SP-B) [2,3] and *ABCA3*, which encodes ATP-binding cassette transporter A3 (ABCA3) typically present as lethal respiratory distress syndrome in neonates [4-6]. Bi-allelic *ABCA3 *mutations [7,8] and mono-allelic mutations of *SFTPC*, the gene encoding surfactant protein-C (SP-C), [9-11] may also cause later-onset, progressive interstitial lung disease spanning from infancy to adulthood. Thyroid transcription factor-1 (TTF-1), also known as NK2 homeobox-1 (NKX2.1) or thyroid-specific enhancer-binding protein (T/EBP), plays a role in embryogenesis and morphogenesis of the lung, brain and thyroid gland [12-14], and regulates the expression of a series of genes implied in surfactant synthesis [15]. TTF-1 haploinsufficiency secondary to deletions or mono-allelic mutations of the *NKX2.1 *gene has been recognized as a rare cause of neonatal or infantile respiratory failure, often associated with congenital hypothyroidism and/or benign hereditary chorea [16-20], referred to as "brain-lung-thyroid syndrome". These genetic disorders are associated with various disruptions of surfactant synthesis and composition [17,21]. Recently, a double stable isotope labeling approach has been described for *in vivo *endogenous surfactant kinetics assessment [22]. We report a patient with severe neonatal respiratory distress syndrome (RDS), recurrent respiratory failure episodes in infancy, pituitary anatomical and functional anomalies, and mild neurological symptoms suggestive of brain-lung-thyroid syndrome, in which extensive surfactant-related gene sequencing failed to identify identified *NKX2.1 *mutations and showed only a previously unreported *ABCA3 *missense mutation carried in heterozygosis.

## Materials and methods

### Patient's clinical history

The infant was a first male child born at 40 weeks of gestation by vaginal delivery, with a one- and five-minute Apgar score of 8 and 9 and normal birth weight. The infant was a first child, and the parents, of east European descent, were non-consanguineous and reportedly healthy. Soon after birth he presented with respiratory distress and hypoxemia, requiring intubation and mechanical ventilation. Since hypoxemia progressed, the infant required three doses of poractant alpha, high-frequency oscillatory ventilation, plus inhaled nitric oxide (iNO) and milrinone. Extubation at seventeen days failed, and mechanical ventilation and iNO were resumed for additional five days. Dexamethasone was added for fourteen days, as well as sildenafil, and the infant was discharged at thirteen weeks in room air. He was readmitted twice in pediatric intensive care unit for respiratory failure and pulmonary hypertension relapse in the course of viral respiratory infections, at the age of four and seven months, and was treated with poractant alpha, dexamethasone, iNO and ventilation for four and nine days respectively. At seven months, a surgical lung biopsy was performed after obtaining parental consent. At one year, failure to thrive, delayed developmental milestones and moderate axial hypotonia became evident. Free thyroxin (FT4) level was 4.5 pg/mL (8.0-19), free triiodothyronine (FT3) was 2.9 pg/mL (1.8-19.0) and thyroid-stimulating hormone (TSH) was 1.25 mUI/L (0.4-4.0). Growth Hormone (GH) levels at baseline and after two separate arginine stimulation tests were <0.5 ng/mL (normal: >10). Cortisol at baseline and after adrenocorticotropic hormone (ACTH) stimulation test was normal. Baseline ACTH was normal. Brain MRI showed an ectopic and hypoplastic pituitary gland, partial optical nerve atrophy, and bilateral occipital white matter injury. Thyroid gland ultrasonography was unremarkable.

### Surfactant-related gene sequence analysis

*SFTPB*, *SFTPC *and *ABCA3 *genes were analyzed by direct sequencing of PCR-amplified products from genomic DNA as published [2,4,23]. Two sets of specific primers were used for amplification of the whole *NKX2.1 *coding and non-coding regions, the sequences of which are available on request. Results were compared to published reference sequences [ENSG00000168878], [ENSG00000168484], [ENSG00000167972] and [ENSG00000136352] respectively. Genomic rearrangements were studied by array chromatin genomic hybridization (aCGH) using a 60 K microarray (Agilent hg19, Agilent Technologies, Santa Clara, CA, USA). Genetic studies were conducted after obtaining parental informed consent. These studies were performed in compliance with the Bambino Gesù Children's Hospital Ethics Committee guidelines.

### Microscopic studies

Sections of formalin-fixed lung tissue were analysed with hematoxylin-eosin (HE), Masson Trichrome (MT), Periodic Acid Shiff (PAS) and Van Gieson (VG) stainings. For immunofluorescence studies, serial lung cryosections were fixed with 4% paraformaldehyde in phosphate buffered saline labeled with monoclonal antibodies against SP-B (Labvision, Fremont, CA), TTF-1 and ABCA3 (clone 13-H2-57, Seven Hills Bioreagents, Cincinnati, OH) or polyclonal antibodies against proSP-B and pro SP-C, transforming growth factor-β1 (TGF-β1) and SMAD3 (Chemicon Inc., Temecula, CA). The immunoreaction was revealed with goat anti-mouse or anti-rabbit Alexa Fluor 488-conjugated immunoglobulins (Molecular Probes, Eugene, OR), or with a goat anti-rabbit Alexa Fluor 555-conjugated antibody (in TTF-1/proSP-C double immunostaining). Nuclear staining was performed with Hoechst 33342 (Molecular Probes). Image acquisition were performed using an Olympus Fluoview FV1000 confocal microscope equipped with FV10-ASW version 1.6 software, and processed with Adobe Photoshop software version 9.0. Ultrathin 1 μ sections obtained from Karnowsky-osmium tetroxide post-fixed and epon-embedded samples contrasted with lead citrate and uranyl acetate were analyzed with a Zeiss 902 transmission electron microscope. For quantitative lamellar body analysis, mean count per cell and diameter measurement were derived from10 random sections at 3000 × magnification picturing single type II cell cross-section. Normal human lung tissue obtained from a lobectomy specimen in a 3-month old infant with congenital cystic adenoid malformation and lung biopsies from five infants with ABCA3 mutations (one homozygous frameshift mutation carrier, one double heterozygous missense mutations carrier and three heterozygous missense mutation carriers) were used as controls after parental consent.

### Surfactant composition and kinetics

After parental informed consent was obtained, the patient received a 24 h IV infusion of 1-^13^C leucine stable isotope, 2 mg/kg/h and a 48 h ^2^H_2_O stable isotope administration, given as 2 mL/Kg bolus followed by 0.125% of total fluid intake, according to a previously published research protocol approved by the conducting institution review board [24,25]. Serial blood, urine and tracheal aspirate (TA) samples were collected for a 48 h period. TA supernatant was separated by centrifugation. Disaturated phosphatidylcholine (DSPC) - the main phospholipid (PL) component in human surfactant- and SP-B were isolated by solid phase extraction and thin layer chromatography. DSPC was quantified by gas-chromatography (GC) and DSPC and SP-B kinetics measured by GC-isotope ratio-mass spectrometry (IRMS) and GC-mass spectrometry (GC-MS) respectively. ^13^C Leucine enrichment at plateau in plasma aminoacids was determined by GC-MS. Deuterium enrichment in urine was determined with a thermal conversion/elemental analyzer coupled with an IRMS to determine ^2^H_2_O plateau enrichment. Fractional synthetic rate was derived from the linear increase of the SP-B ^13^C leucine and of the DSPC ^2^H-palmitate respectively, as published. Six infants with gestational age >37 weeks, intubated and ventilated for conditions unrelated to parenchymal lung disease, who underwent the same protocol after parental consent, were used as controls.

## Results

### Morphology

**Figure 1:**
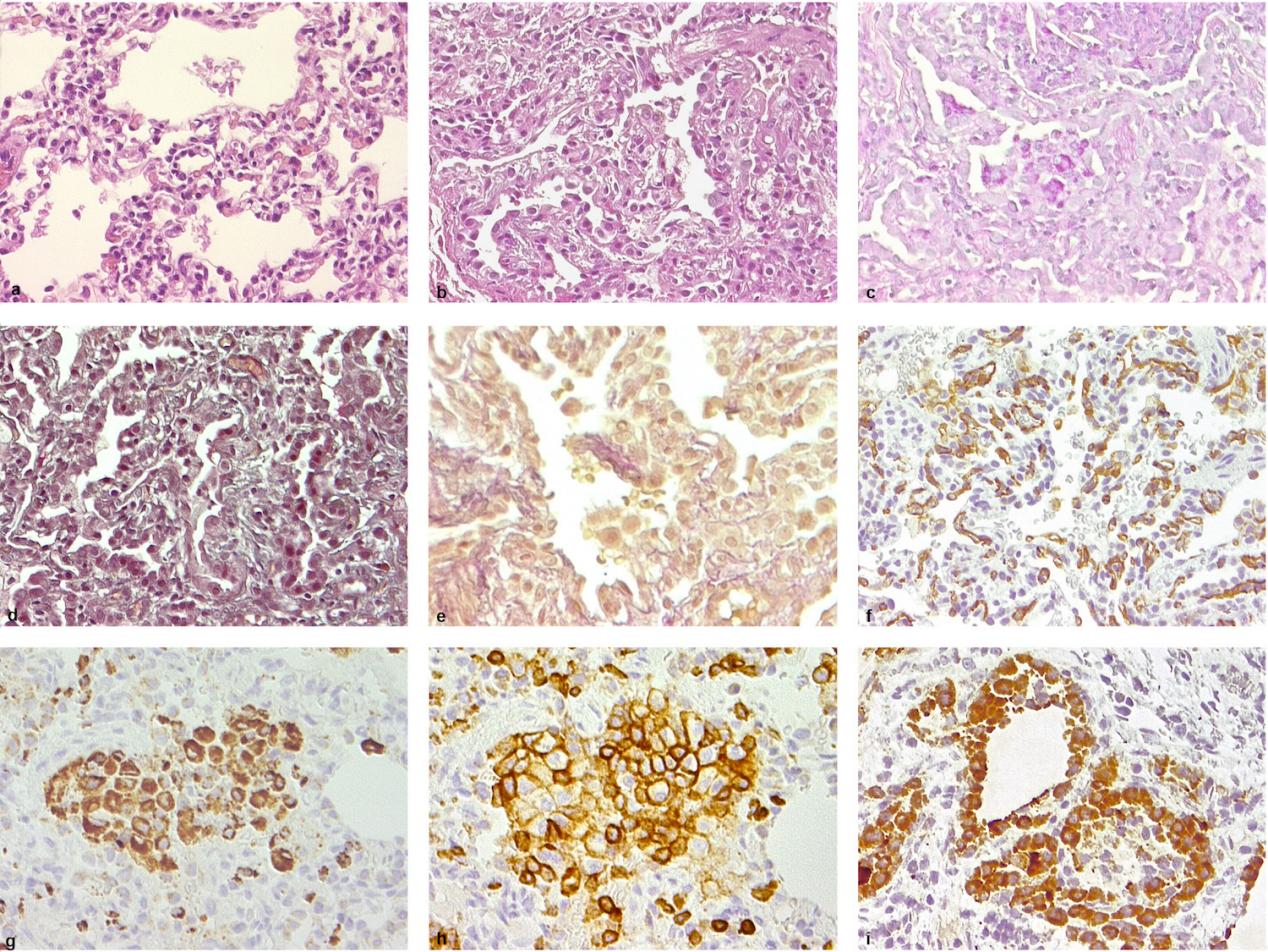
**Lung tissue morphology**. a: normal control (healthy lung tissue obtained from lobectomy in a 1-month old infant with congenital cystic adenoid malformation), optical microscopy, 20 ×; b-i: patient's lung tissue obtained by open-chest biopsy at 7 months. a: Normal lung tissue, HE, 20 ×; b: low-power microscopy shows interstitial thickening, alveolar type II cell hyperplasia and intra-alveolar amorphous material (HE 20 ×); c: sparse intracellular and intra-alveolar proteinaceous material accumulation (PAS, 20 ×); d: Diffuse interstitial fibrosis (MT, 20 ×); e: Small collagen fiber deposition in the interstitium (VGFE, 20 ×); Regular density and distribution of pulmonary capillary vessels (factor VIII, 20 ×); g: Higher magnification shows leukocyte intra-alveolar accumulation and interstitial infiltration (CD45, 40 ×); h: intra-alveolar cells mostly correspond to macrophages (CD68, 400 ×); i: alveolar epithelial lining consists of hyperplastic type-II cells (proSP-B, 40 ×).

**Figure 2:**
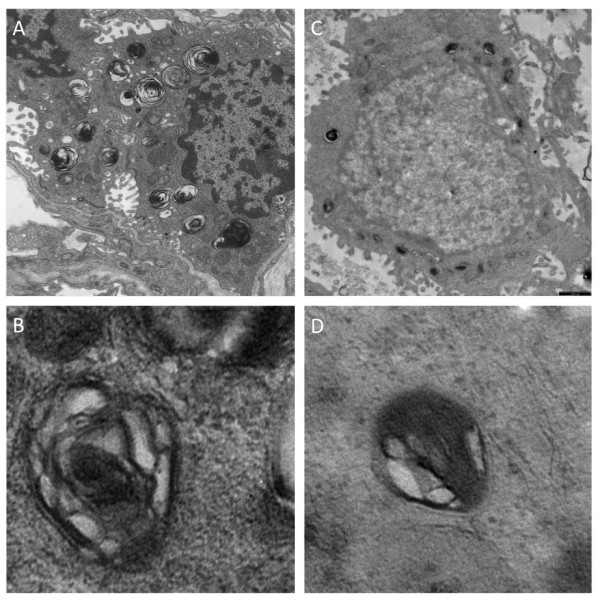
**Alveolar type II cell ultrastructure**. Transmission electron microscopy of lung tissue. A. normal lung tissue of a 5 month-old infant obtained from lobectomy for congenital cystic adenoid malformation showing a type II cell with numerous lamellar bodies filled with concentric pseudomyelin membranes, magnification 3000 ×. B. Detail of one lamellar body, 8000 ×. C. lung tissue form the patient's biopsy, showing a type II cell with smaller, denser lamellar bodies. D. Detail of one lamellar body with poorly structured content, Magnification bar: 1 μm.

Lung microscopy revealed diffuse interstitial thickening with thin collagen fiber deposits on MT- and VG-stained sections, and with predominantly lymphomonocytic (CD45 positive) cell infiltrates plus some neutrophils and eosinophils, alveolar type II (proSP-B positive) cell hyperplasia and abundant clusters of intra-alveolar macrophages (CD68-positive) with a foamy, PAS-positive cytoplasm, a pattern corresponding to desquamative interstitial pneumonitis (DIP). Alveolar spaces were normal-sized, and, within the limits of the sample, bronchiolar architecture was unremarkable. Only minor intra-alveolar amorphous material was seen (PAS), which excluded alveolar proteinosis. Arterioles did not show significant signs of remodeling, and the pulmonary capillary bed was quantitatively and morphologically well represented (CD31) (figure 1). On transmission electron microscopy, lamellar bodies count per cell were similar to control (15.3 ± 3.1 vs. 14.4 ± 4.0) but their diameter was smaller (618 ± 98 vs. 852 ± 135 nm), with few electron-dense concentric membranes and a denser central core similar as those found in ABCA3 deficiency [26,27] (figure 2).

### Molecular genetics

*SFTPB *sequencing revealed the presence of the homozygous c2052 C>A and the heterozygous c2619 T>C polymorphisms. *SFTPC *sequencing showed the presence of the homozygous c2772 A>G and c2643 C>G polymorphisms. *ABCA3 *sequencing showed a mono-allelic variation, c3381 T>C, leading to the aminoacidic sequence change L941P, not previously reported, which was carried by the father and was not present in 100 control alleles, hence to be considered a novel heterozygous missense mutation. On *NKX2.1 *sequencing, four common variants were present: rs76977831, rs77021012, rs117543880, rs117216249. In addition we found a insertion variant in the 3'UTR-coding region, 1636_1637 ins AC, but it was found to be present in the proband's mother and in 3 out of 60 alleles from unaffected infants, one being homozygous carrier for this variant, which therefore doesn't appear to be disease-causing. The aCGH analysis did not reveal copy number variations in the NKX2.1, ABCA3, SFTP-B and SFTP-C loci.

### Surfactant-related protein expression

**Figure 3:**
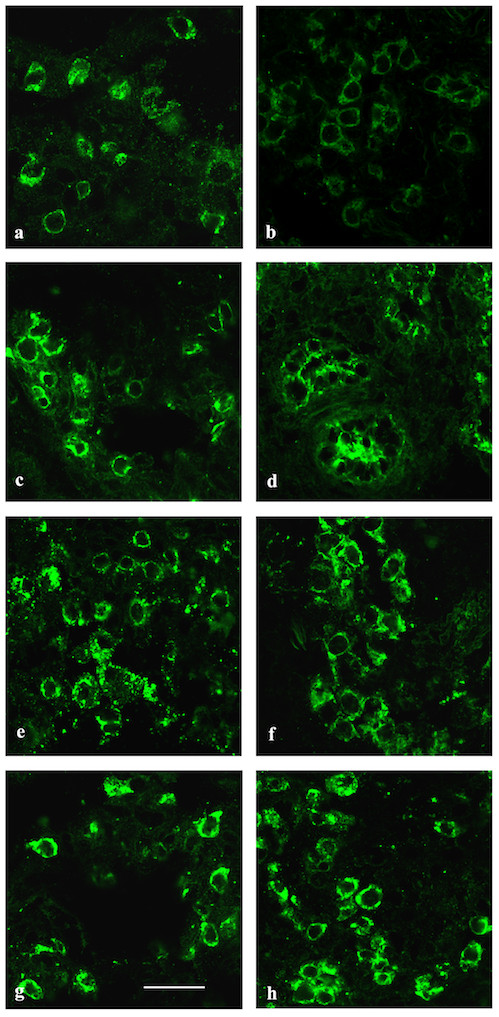
**Surfactant-related protein expression**. Confocal microscopy of lung biopsies from control (a, c, e, g) and patient (b, d, f, h), immunolabeled with antibodies against ABCA3 (a-b), pro SP-B (c-d), SP-B (e-f), pro SP-C (g-h) antibodies. ABCA3 labeling showed a faint and homogeneous reduction in the type II cell population in patient compared to control, whereas pro SP-B, SP-B and pro SP-C protein expression was similar. Magnification bar: 20 μm.

**Figure 4:**
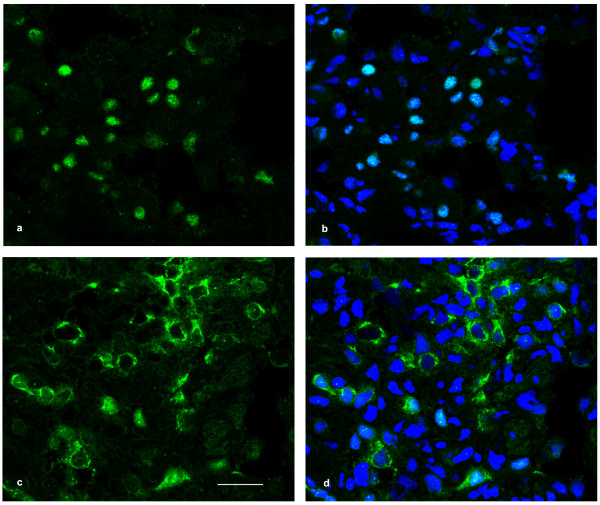
**TTF-1 expression**. Lung tissue immunolabeled with anti-TTF-1 antibody (green) and nuclear labelling (Hoechst 33342, blue), confocal microscopy. a-b: TTF-1 expression in normal lung is confined to nuclear districts. c-d: TTF-1 protein distribution in patient's lung is markedly decreased in alveolar type II cell nuclei (c) and predominantly confined in their cytoplasm. Magnification bar: 20 μm.

ABCA3 expression was moderately decreased, while proSP-B, mature SP-B and proSP-C expression were similar in the patient compared to control (figure 3). In the control, as described in the literature [28], TTF-1 was almost exclusively expressed in the nuclei of alveolar type II cells, as shown by co-expression of pro-SP-B (not shown), whereas in the patient, it appeared mostly confined to the cytoplasm and barely detectable in the nucleus (figure 4). This pattern was not found in the five *ABCA3 *mutation controls (not shown). TGF-β1 and SMAD3 expression resulted similar to controls (not shown).

### Phospholipid and SP-B composition and metabolism

The surfactant kinetic study was conducted at the age of 8 month, while the patient was admitted the pediatric intensive care for a respiratory failure relapse. There was a marked (~50%) reduction of DSPC (29.8 vs. 56.1 ± 12.4% PL) in the patient's TA compared to controls values from our laboratory (mean ± standard deviation). DSPC fractional synthesis rate was significantly increased in the patient compared to controls (12.4 vs. 6.3 ± 0.5%/day), while SP-B synthesis rate was markedly reduced (43.2 vs. 76.5 ± 24.8%/day) (figure 3).

## Discussion

TTF-1 is a transcription factor accepted as a master regulator of foregut and forebrain structures development. Complete TTF-1 absence in the *NKX2.1 *null mouse leads to non-viable progeny with defective lung organogenesis and bronchial branching, absent thyroid gland, forebrain anomalies and absent pituitary [12,13]. In the lung, TTF-1 is expressed in the alveolar epithelium and is required for type II cell differentiation and surfactant protein expression. Pulmonary pathology in human subjects with TTF-1 haploinsufficiency is characterized by mixed features of lung development impairment (reduced airway generations and radial alveolar count, distal bronchiolar cysts) and surfactant homeostasis disruption (focal alveolar septal fibrosis, alveolar type II cell hypertrophy and clusters of alveolar macrophages) [19,29]. Cytoplasm-restricted TTF-1 expression in type-II cells has not been, to our knowledge, observed in subjects with diffuse lung disease. In our case, optical and ultrastructural morphology are more indicative of a surfactant defect, while no developmental abnormalities are observable [30]. Complete TTF-1 cytoplasmic restriction would be expected to abolish homeodomain nuclear transcription, which is not compatible in this case. Some degree of residual TTF-1 nuclear targeting may explain the observed phenotype. A similar pattern has been reported in an *in vitro *mutagenesis study reproducing a human *NKX2.1 *mutation [31]; hence it could represent an alternative molecular mechanism in certain cases of TTF-1 haploinsufficiency. TTF-1 cytoplasmic trapping was also observed *in vitro *in human lung cultures exposed to phorbol ester -a nuclear translocation-blocking compound [32]- or to TGF-β1 [33]; in these experiments, cytoplasmic trapping of TTF-1 -a known inductor of *SFTPB *and *SFTPC *genes-resulted in *SFTPB *down-regulation [34]. For this reason we studied TGF-β1 and SMAD3 expression, but it resulted similar to controls. Moreover, a more pervasive defect of the nuclear translocation machinery of the cell appears very unlikely, since it would affect many nuclear proteins and would probably not be viable. Our patient had a 50% reduction of SP-B synthesis rate, a finding consistent with the fact that SP-B and SP-C content is altered in tracheal aspirates of patients with TTF-1 deficiency [16,17]. These data suggest that decreased SP-B synthesis due to defective TTF-1 nuclear translocation contributed to the respiratory phenotype.

We also showed a marked reduction of DSPC content in the alveolar fluid, despite an increased fractional synthesis rate. Such a finding has been described in TA of patients with ABCA3 deficiency [35] and in ABCA3-deficient mice [36]. *ABCA3*, which encodes a transmembrane phospholipid transporter critical for intracellular surfactant assembly and packaging [36], is also a target gene for TTF-1 [37]. Indeed, ABCA3 expression appeared decreased in our patient. Moreover, he carried a novel *ABCA3 *missense mutation in heterozygosis. Since this variation has not been previously described, mutagenesis studies would be necessary to fully assess its relevance; however, its location in the 7^th ^transmembrane domain coding sequence suggests it potentially affects protein function [38]. Mono-allelic *ABCA3 *missense mutations have been reported as modifiers of other genetic surfactant defects [39,40] and may increase RDS severity in susceptible individuals [41]. Overall we can speculate that partial ABCA3 insufficiency due to the combined effects of TTF-1 cytoplasmic trapping and the missense *ABCA3 *mutation further contributed to respiratory phenotype, causing a latent surfactant homeostasis disorder with exacerbation under stress circumstances such as viral infection.

Although the clinical phenotype and immunolocalization studies strongly suggest a TTF-1 genetic defect leading to partially defective nuclear targeting, we were not able to demonstrate any mutation or deletion affecting coding and non-coding regions of the NKX2.1 gene. We cannot formally exclude post-transcriptional anomalies or variations not accessible by the techniques applied in this case, and even if our data do not support a role of TGFβ in TTF-1 sequestration, we cannot exclude anomalies in other genes interfering with TTF-1 nuclear translocation. Since we were unable to show TTF-1 trapping in other homozygous or heterozygous ABCA3 mutation carriers in our hands, and since no data in the literature suggest that ABCA3 affects *NKX2.1 *expression, it is unlikely that the TTF-1 targeting defect is secondary to the ABCA3 mutation.

TTF-1 plays an essential role in central nervous system morphogenesis. To our knowledge, brain imaging and histology studies in subjects affected by TTF-1 haploinsufficiency are usually negative or nonspecific [19,20]. However, heterozygous interstitial chromosome 14q deletions encompassing *NKX2.1 *may be associated with pituitary hypoplasia and ocular anomalies [42-44], and in animal studies TTF-1 is critical for forebrain and pituitary embryogenesis [12]. Hence, in our case pituitary malformation is presumably caused by TTF-1 signaling disruption, leading to central hypopituitarism and GH deficiency. This pattern differs from the peripheral hypothyroidism typically associated with TTF-1 haploinsufficiency.

In summary we report a complex surfactant homeostasis disorder caused by a TTF-1 defect of unknown origin, not previously described, combined to a novel heterozygous *ABCA3 *mutation in a patient with brain-lung-thyroid syndrome. Although this compound genetic disorder may remain unique to this kindred, it highlights the importance of conducting extensive morphological, molecular and genetic studies in patients with unexplained diffuse lung disease in order to establish solid genotype-phenotype correlations and identify new genetic defects in this highly heterogeneous and under-recognized group of diseases.

## Abbreviations

SFTPB: surfactant protein-B gene; SP-B: surfactant protein-B; ABCA3: adenosine triphosphate-binding cassette transporter A3; SFTPC: surfactant protein-C gene; SP-C: surfactant protein-C; TTF-1: thyroid transcription factor-1; NKX2.1: NK2 homeobox-1; T/EBP: thyroid-specific enhancer-bnding protein; RDS: respiratory distress syndrome; iNO: inhaled nitric oxide; FT4: free thyroxin; FT3: free triiodothyronine; TSH: thyroid-stimulating hormone; GH: growth hormone; ACTH: adrenocorticotropic hormone; aCGH: array chromatine genomic hybridization; HE: hematoxylin-eosin; MT: Masson trichrome; PAS: periodic acid Schiff; proSP-B: surfactant apoprotein-B; proSP-C: surfactant apoprotein-C; TGF-β1: transforming growth factor-β1; TA: tracheal aspirate; DSPC: disaturated phophatidylcholine; PL: phospholipids; GC: gas chromatography; IRMS: isotope ratio-mass spectrometry; MS: mass spectrometry.

## Competing interests

The authors declare that they have no competing interests.

## Authors' contributions

DP carried out molecular genetic studies and analysis, and co-drafted the manuscript; SP carried out protein expression immunofluorescence studies, plus confocal and electronic microscopy; CT directed and collected clinical investigations and contributed to draft the manuscript; RB carried out optical and electronic microscopy studies; FM contributed to clinical investigations and contributed to draft the manuscript, MS contributed to clinical investigations; VC contributed to kinetic studies and data interpretation, PC carried out kinetic studies and contributed to draft the manuscript; OD conceived the study, carried out data analysis and drafted the manuscript. All authors read and approved the final version.
